# Periodontal Plastic Surgery to Improve Aesthetics in Patients with Altered Passive Eruption/Gummy Smile: A Case Series Study

**DOI:** 10.1155/2012/837658

**Published:** 2012-09-30

**Authors:** Francesco Cairo, Filippo Graziani, Lorenzo Franchi, Efisio Defraia, Giovan Paolo Pini Prato

**Affiliations:** ^1^Department of Periodontology and Implant Dentistry, Tuscan School of Dental Medicine, University of Florence-Siena, 50121 Florence, Italy; ^2^Department of Surgery, Unit of Dentistry and Oral Surgery, University of Pisa, Pisa, Italy; ^3^Department of Orthodontics, University of Florence, 50121 Florence, Italy

## Abstract

Altered passive eruption/gummy smile is a common challenge in patients requiring aesthetic treatment. A specific surgical protocol was designed and tested in patients with altered passive eruption. Standardized preoperative X-rays were used to assess crown length at baseline and to place submarginal incisions. Osseous respective therapy was performed to achieve biological width. Clinical outcomes were recorded 6 months after surgery. Eleven patients with a total of 58 teeth were treated with flap surgery and osseous resective therapy at upper anterior natural teeth. At the last followup, a significant and stable improvement of crown length was obtained when compared to the baseline (*P* < 0.0001). All patients rated as satisfactory in the final outcomes (final VAS value = 86.6). In conclusion, this study showed that periodontal plastic surgery including osseous resection leads to predictable outcomes in the treatment of altered passive eruption/gummy smile: A careful preoperative planning avoids unpleasant complications and enhances postsurgical stability of the gingival margin.

## 1. Introduction

A pleasant smile is considered a symbol of beauty and well being in the modern society. A variety of factors including teeth form/position and gingival tissue levels may influence the overall smile aesthetics [1, 2]. In the last decade a great interest was focused on plastic periodontal surgery as a reliable tool to enhance esthetics. A large number of randomized trials showed that the combination of coronally advanced flap and connective tissue graft was associated with the highest probability to obtain complete root coverage (CRC) in the treatment of gingival recession [3]. Furthermore, quality improvement in root coverage outcomes is currently advocated. CRC with soft tissue integration along with the adjacent tissue, including good color match and absence of scars, is now considered as the final goal [4, 5].

Along with gingival recessions, the excessive gingival display during smiling is a frequent condition impairing smile esthetics [2]. This condition is frequently related with an altered passive eruption (APE) of teeth mostly due to developmental or genetic factors that may lead to the persistence of excessive soft tissue amount over the enamel surface. In fact, after the completion of active eruption phase, a passive eruption with an apical migration of soft tissue generally occurs. During this process the epithelial junction apically shifts to the cemento enamel junction (CEJ) level, thus reaching a final position of the gingival margin slightly coronal to the CEJ. More severe cases may be also associated with hyperplastic growth of maxillary skeletal base [2]. The prevalence of APE is reported to be approximately 12% considering more than 1,000 adult patients with mean age of 24 years [6]. This condition may create esthetic concerns due to the display of excessive quantity of gingival tissue at upper anterior teeth when smiling (gummy smile).

APE has been subclassified into 2 types [6]. Type I APE is characterized by an excessive amount of attached gingiva with shorter crowns while type 2 is a gummy smile associated with a normal gingival dimension. Two possible subclasses were also suggested, A and B, depending on the relationship of the osseous crest to the CEJ of the tooth (OC-CEJ). In subcategory A, OC-CEJ is greater than 1 mm leading to adequate space for the insertion of the connective tissue attachment in the root surface, while in subcategory B this space is minimal and does not allow a correct biological width [7].

Possible treatment options of APE type I include gingivectomy and apically positioned flap (APF) plus osseous respective surgery [2]. Conversely, APE type 2 showing excessive growth of the maxillary process generally implies a multidisciplinary treatment plan including prosthodontics, orthodontics, and periodontal surgery [2]. Type 1 APE is a challenge for the periodontist since bone resection in upper natural anterior teeth is a risky and demanding procedure. On one hand, excessive bone resection may lead to residual gingival recession, on the other hand a limited resection and flap management may determine only a partial resolution of APE. Furthermore, a coronal regrowth of the gingival margin following APF may frequently happen reducing the length of postsurgical clinical crowns. Therefore, the lack of a properly planned surgical procedure may cause an esthetic failure when treating APE.

The aim of this study is to test a surgical protocol including flap surgery and osseous resective surgery to improve aesthetics in patients with APE at upper anterior teeth.

## 2. Materials and Methods

Patients were enrolled at the Department of Periodontology, University of Florence, Italy, between February 2010 and October 2010. The department committee approved the study protocol. Nonsmoking patients, showing no sign of periodontitis, were selected and enrolled in this study. Inclusion criteria were the following:

1. age ≥ 18 years;
2. absence of systemic diseases;
3. full mouth plaque scores (FMPS) and full mouth bleeding scores (FMBS) <20%;
4. no site with probing depth >3 mm;
5. aesthetic request due to type 1 APE condition.

Patients showing teeth with malposition and/or alteration in crown morphology were excluded from the study. All participants gave a written consent to participate to the study.

### 2.1. Pretreatment Measurements

**Figure 1:**
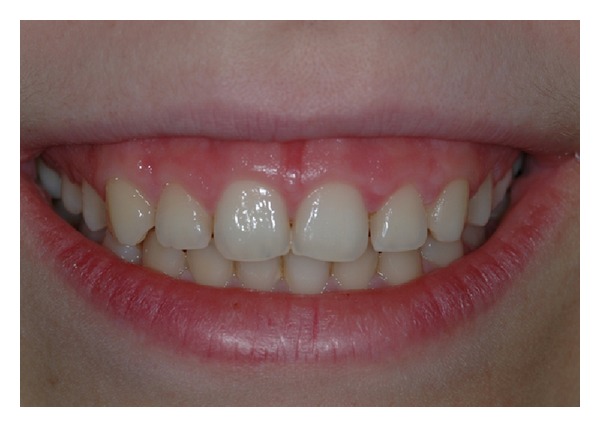
Pretreatment smile of patient n.5.

**Figure 2:**
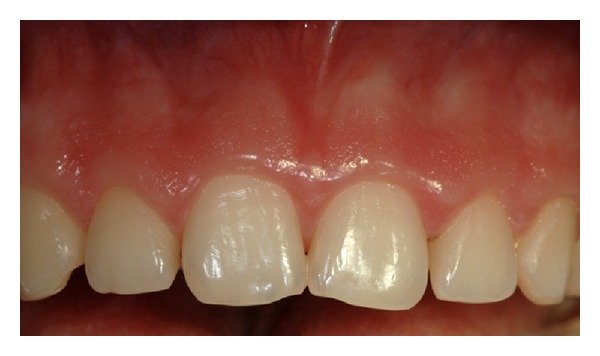
Pretreatment view of upper anterior teeth.

**Figure 3:**
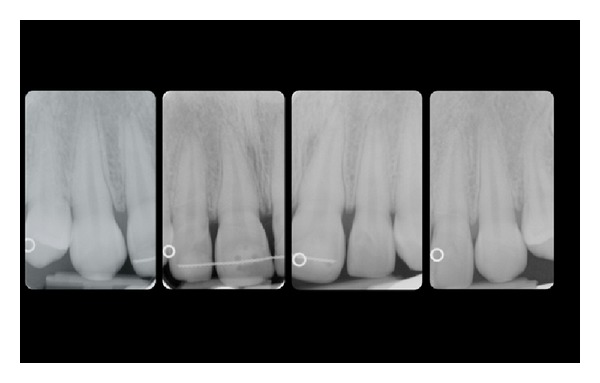
Periapical X-ray of the upper anterior teeth to plan sub-marginal incision (see test for explanation).

A calibrated examiner not involved in the surgical procedures took clinical measurements. After pretreatment clinical evaluation (Figures 1 and 2), the following measurements were taken on a periapical, standardized X-ray of the involved teeth (Figure 3):

- length of the crown on X-ray (L-rx), that is, distance between the incisal margin (IM) and the CEJ;
- width of the crown on X-ray (W-rx), that is, distance between mesial and distal angle at the incisal margin.

In addition, the following clinical measures were taken using a periodontal probe (UNC-15 periodontal probe):

- length of the crown at baseline (IM-GM0), that is, distance between the incisal margin (IM) and gingival margin at baseline (GM0);
- width of the clinical crown at the baseline (W-cl), that is, distance between mesial and distal angle at the incisal margin.

The real clinical length of the crown (L-cl) is then calculated in a presumptive manner (L-cl = (L-rx × W-cl) ÷ W-rx) as CEJ was not clinically visible. L-cl estimates of teeth to be treated were then used as a reference point for surgical planning. Accordingly, the level of incisions at the surgical site were placed submarginally, 0.5 mm coronal to the estimated CEJ level. 

### 2.2. Surgical Procedures

After the identification of CEJ level the following surgical steps were performed (all phases are pictured in Figures 3–10).

**Figure 4:**
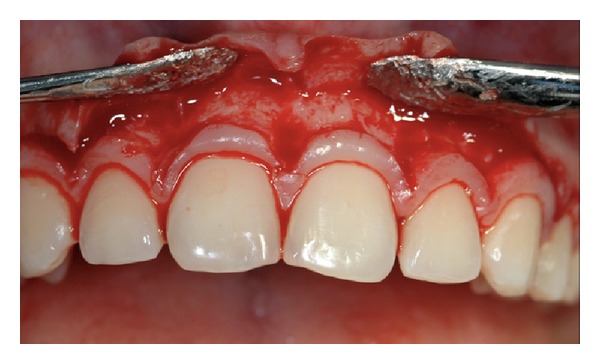
Submarginal incision 0.5 mm coronal to the CEJ level.

**Figure 5:**
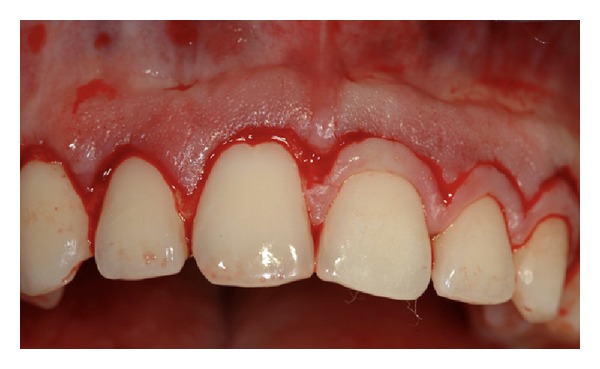
Elimination of secondary flap. Note the immediate increase in crown length.

**Figure 6:**
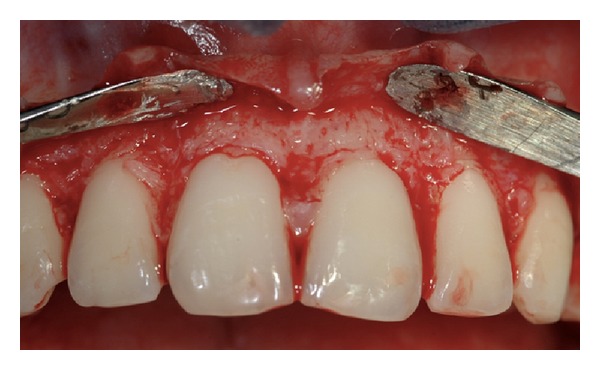
Bone level after flap reflection.

**Figure 7:**
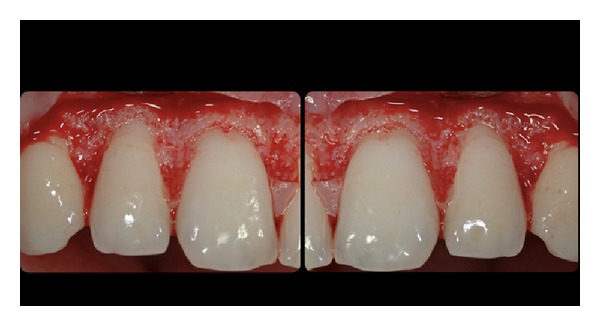
Bone crest after osseous resective surgery. Approximately 1 mm of root surface was exposed.

**Figure 8:**
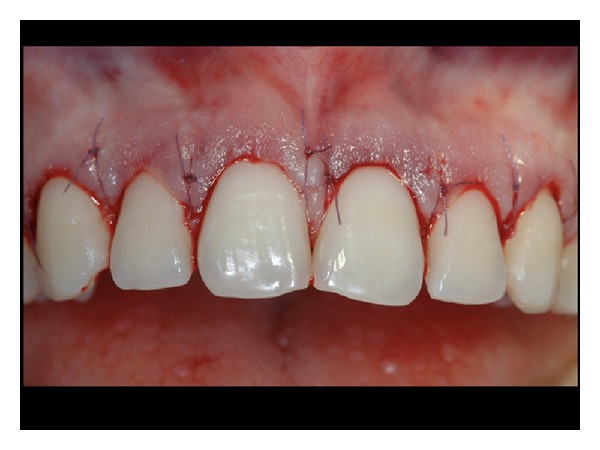
Final suture of the flap at the preestablished level.

1. Following the local anesthesia, a submarginal incision, approximately 0.5 mm coronal to the calculated CEJ level, was performed at each treated tooth using a 15 c blade. Only the buccal site was involved in the surgical procedure (Figure 4). Care was taken to completely maintain interproximal papillae *in situ*. The secondary flap was then removed after intrasulcular incisions using a sharp curette (Figure 5).
2. A full thickness flap was then elevated using a small periosteal elevator (Figure 6). Bone exposure was limited to 4-5 mm. As a rule the flap was elevated until the mucogingival junction.
3. Intrasurgical measurement of the distance between bone crest and CEJ was then performed. When the distance was <1 mm, a gentle osseous resection was accomplished to create a scalloped bone profile with at least 1 mm of distance to the CEJ. Ideally, the osseous crest was shaped parallel to the CEJ. Osteoplasty was performed when necessary. The exposed root surface was then carefully planned to eliminate any residual inserted fibres at the buccal treated sites only (Figure 7). Care was taken to preserve the attachment apparatus at interproximal site.
4. The flap was then sutured at the preestablished level, slightly coronal to the CEJ level, to obtain a primary closure using of interrupted resorbable sutures (Figure 8).

After suture, the new length of the clinical crown was assessed as the distance between incisal margin and flap margin after suture (IM-GM1). All surgical procedures were performed by the same experienced periodontist (FC).

### 2.3. Postsurgical Procedures

**Figure 9:**
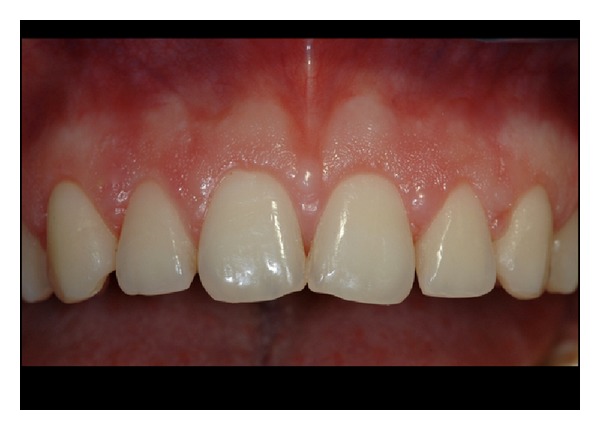
Soft tissue healing 6 months after surgery.

**Figure 10:**
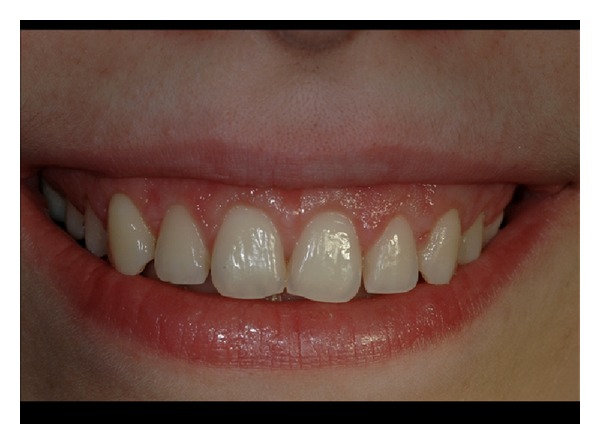
Final patient smile after healing.

**Figure 11:**
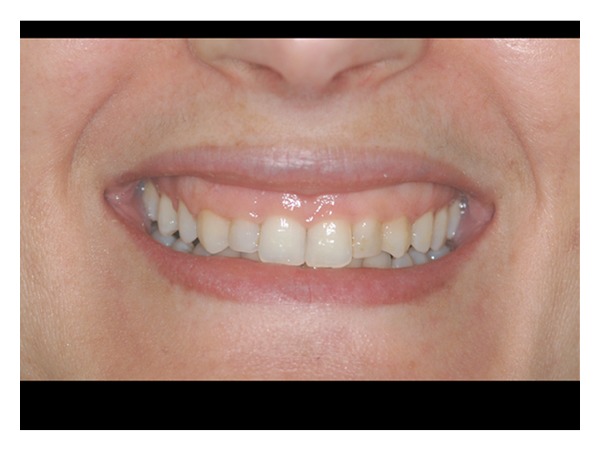
Pretreatment view of patient n.9.

**Figure 12:**
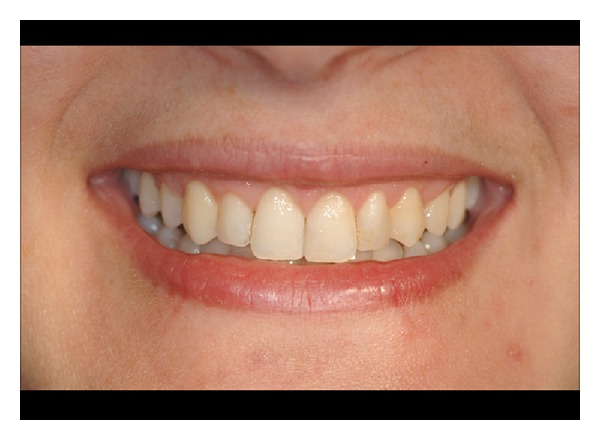
Posttreatment view of patient n.9.

Following the completion of the surgical procedure, all patients received appropriate analgesic treatment and advised to avoid any trauma or tooth brushing in the treated area for 2 weeks; 0.12% chlorhexidine rinses were also prescribed. Sutures were removed 7 days following surgical procedures. Professional tooth cleaning consisting of supragingival prophylaxis with a rubber cup and 1% Chlorhexidine gel application was performed at 2-weeks and 4-weeks followup. All patients were maintained in a supportive periodontal-care program at monthly intervals until the final followup (6 months) (Figures 9-10), when the final length of the clinical crown was assessed as the distance between incisal margin and gingival margin (IM-GM2) (Figures 11 and 12, case n.9 is shown). At the final followup patient satisfaction was evaluated with visual analogue scale (VAS) from 0 to 100.

### 2.4. Statistics

Data were entered into an Excel (Microsoft office 2007) database and were proofed for entry errors. The database was subsequently locked, imported into SPSS for Windows (SPSS Inc., version 16.0) formatted, and analyzed. Data were summarized as means and 95% confidence intervals. Analysis was performed on both subject and tooth level. Subject-level analysis was performed by computing a subject-level variable. The significance of the treatment on clinical crown length was estimated by analysis of variance (ANOVA). In order to identify differences between various groups, posthoc analysis was performed with Fisher's least significant difference (LSD) test. An intention-to-treat, last observation carried forward analysis was performed. Significance was attributed when *P* was <0.05 (Tables 1 and 2).

## 3. Results

A total of 11 patients were enrolled in this study (10 female and 1 male). The mean age was 24.9 ± 6.5 years. A total of 58 upper maxillary teeth showing altered passive eruption were treated: 21 central incisors, 12 lateral incisors, 19 canines, and 6 first premolars. 

The mean length of the clinical crown at the baseline was 8.5 ± 0.9 mm. After flap suture, the new mean length of the clinical crown assessed as the distance between incisal margin and flap margin after suture was 10.2 ± 0.7 mm (*P* < 0.0001). Healing was uneventful in all treated sites with no flap dehiscence or lack of primary closure. All patients were satisfied of the final clinical outcomes.

Patients maintained throughout the study a high level of plaque control (FMPS and FMBS <20%). The final mean length of the clinical crown was 10.1 ± 0.7 mm. No gingival recession and no sites with probing depth >3 mm was detectable at the treated sites after 6 months. All patients rated as satisfactory in the final outcomes (VAS = 86.8, ranging from 65 to 100).

Statistical analysis showed a significant difference in term augmentation in clinical crown length compared with baseline (*P* < 0.0001) at both patient and site levels. On the other hand, no statistically significant difference was detected when comparing the mean length of clinical crown after suture to the mean final crown length at both patient (*P* = 0.482) and tooth site (*P* = 0.498). 

## 4. Discussion

Our data indicated that a careful preoperative planning might avoid unpleasant complications and enhance postsurgical stability of the gingival line in the surgical treatment of APE. In particular, identification of CEJ level allows a surgical resection that appears (i) to be stable over time and (ii) optimize esthetics. 

An increasing stringent demand for improvement of esthetics is part of the current periodontal practice. Esthetic treatment of a smile line is often a multifaceted scenario where teeth, periodontal tissues, and lip position interact. Some attempts are reported in literature to define the factor influencing smile esthetics. The average smile exhibits approximately the full length of the maxillary anterior teeth, with an incisal curve of the teeth parallel to the inner curvature of the lower lip [8]. In this clinical scenario the upper teeth until premolars are usually displayed. 

Clinical parameters to restore dental and gingival esthetics in the maxillary anterior identified the zenith position of the gingival contour as a key factor. Current standards in the average smile suggest locating the gingival contour at cuspids at similar level or slightly apical than at central incisors, while a more coronal gingival contour is suggested for lateral incisors [9]. 

The treatment of APE may represent a challenge for clinician; in fact, excessive amount of bone resection may lead to final residual gingival recession, while a poor bone resection/flap management may determine a partial resolution of APE. In this clinical trial a well-defined surgical procedure to correct gummy smile was applied. In order to plan correctly the position of submarginal incision, a preliminary study on X-ray was performed calculating the real clinical crown dimension. Care was taken to elevate the flap until the MGJ, thus achieving a good control of the surgical gingival margin and then simplifying suture and flap stabilization. Furthermore, in this study osseous resective surgery as part of periodontal plastic surgery was performed to allow obtaining a stable apical shift of the gingival margin. Recent observations [10] suggested that biological width might extensively vary in different patients leading to final amount of supra-alveolar tissue (ST) different than the standard of 2.73 mm reported by Gargiulo [11]. In fact, ST may be different in patients but also in the same patient at different sites, with significant differences comparing tooth types (anterior versus posteriors teeth) or tooth surfaces (facial/lingual versus interproximal sites) [11]. In this study care was taken to create 1 mm of root exposure in order to favorite proper change in the gingival margin position. Final outcomes showed that the used procedure was effective with no residual gingival recession, stable improvement of crown length compared to the baseline (*P* < 0.0001), and high patient satisfaction (mean final VAS = 86.8). 

A classical paradigm in periodontal surgery is that soft tissue healing is influenced by the position of gingival margin with respect to the bone crest: a study on crown lengthening procedure showed that at least 3 mm of coronal regrowth of gingival margin 6 months after surgery is expected when the flap is sutured at the bone crest [12]. In this study the final position of the gingival margin was apical to the baseline position but coronal to the bone crest. This unusual type of apically positioned flap may at least in part explain the minimal regrowth of the gingival margin after healing compared with the flap position at time of suture. On the other hand, the reached amount of clinical crown lengthening (approximately 1.5 mm) was statistically significant (*P* < 0.0001) at last followup compared to baseline at both patients and tooth site. This implies that the overall procedure is effective to obtain an effective, stable apical shift of the gingival margin position in treating gummy smile. 

Literature concerning treatment of gummy smile is generally anecdotal and sparse with no data supported by statistical analysis. Case reports generally described the use of periodontal surgery with no clear difference between gi sungivectomy and osseous resective surgery. Multidisciplinary treatment plans including prosthodontics and orthodontics are generally suggested for cases showing excessive growth of the maxillary process [2]. In this case series, the reported outcomes showed that osseous resection is strongly recommended to obtain stable improvement of the smile.

## 5. Conclusion

This study showed that periodontal plastic surgery including osseous resection leads to predictable outcomes in the treatment of altered passive eruption/gummy smile. A careful preoperative planning avoids unpleasant complications and enhances postsurgical stability of the gingival margin.

## Figures and Tables

**Table 1:** Values of clinical crown length (L-cl) at patient level.

	L-cl (mm)	Dev. St.	Difference from previous measurement (mm)	*P* value from baseline
Baseline	8.5985	0.49556	NA	
Postoperative	10.2670	0.39185	−1.73276	*P* < 0.0001
6 months	10.1383	0.37434	0.10345*	*P* < 0.0001

*Difference postoperative 6 months, not significant.

**Table 2:** Values of clinical crown length (L-cl) at tooth level.

	L-cl (mm)	Dev. St.	Difference from previous measurement (mm)	*P* value from baseline
Baseline	8.4828	0.90304	NA	
Postoperative	10.2155	0.76153	−1.73276	*P* < 0.0001
6 months	10.1121	0.78942	0.10345	*P* < 0.0001

*Difference postoperative 6 months, not significant.
